# The Yellow Gorgonian *Eunicella cavolini*: Demography and Disturbance Levels across the Mediterranean Sea

**DOI:** 10.1371/journal.pone.0126253

**Published:** 2015-05-05

**Authors:** Maria Sini, Silvija Kipson, Cristina Linares, Drosos Koutsoubas, Joaquim Garrabou

**Affiliations:** 1 Department of Marine Sciences, Faculty of Environment, Univeristy of the Aegean, Mytilene, Lesvos, Greece; 2 Department of Biology, Faculty of Science, University of Zagreb, Zagreb, Croatia; 3 Departament d’Ecologia, Universitat de Barcelona, Barcelona, Catalonia, Spain; 4 Institut de Ciències del Mar, CSIC, Barcelona, Catalonia, Spain; 5 CNRS/INSU, IRD, Aix-Marseille Université, Université du Sud Toulon Var, Mediterranean Institute of Oceanography (MIO), Marseille, France; Università di Genova, ITALY

## Abstract

The yellow octocoral *Eunicella cavolini* is one of the most common gorgonians thriving in Mediterranean hard-bottom communities. However, information regarding its distribution and ecology in several parts of the Mediterranean is lacking, while population trends and conservation status remain largely unknown. We investigated 19 populations of *E*. *cavolini* over three representative geographic regions: the NW Mediterranean, CE Adriatic, and N Aegean. Focusing on the upper bathymetric range of the species (<40 m), data were collected on the populations’ upper depth limit, density, colony height, and extent of injury. A three-level hierarchical sampling design was applied to assess the existence of spatial patterns, using: a) regions (located thousands of km apart), b) localities within regions (tens to hundreds of km apart), and c) sites within localities (hundreds of m to a few km apart). In the NW Mediterranean and CE Adriatic, the upper distribution limit was at depths ≤15 m, whereas in the N Aegean most populations were found deeper than 30 m. Population density ranged between 4.46-62 colonies per m^2^, while mean colony height was 15.6±8.9 SD cm with a maximum of 62 cm. The NW Mediterranean sites were characterized by dense populations dominated by small colonies (<20 cm), periodic recruitment, and low proportion of large gorgonians (>30 cm). The CE Adriatic displayed intermediate densities, with well-structured populations, and continuous recruitment. In the N Aegean, most populations presented low densities, high proportion of large colonies, but low number of small colonies, signifying limited recruitment. Disturbance levels, as a function of extent and type of injury, are discussed in relation to past or present human-induced threats. This work represents geographically the most wide ranging demographic study of a Mediterranean octocoral to date. The quantitative information obtained provides a basis for future monitoring at a Mediterranean scale.

## Introduction

Demographic studies provide valuable information regarding the ecology of long-lived benthic octocorals (e.g. [1–4]). The size structure of a population reflects how key life history parameters, such as growth, reproduction and mortality, have been shaped through its interaction with the surrounding environment and the local stressors [5–8]. Similarly, knowledge on the distribution and population dynamics of a species over large spatial scales enables a more comprehensive understanding of its ability to persist under variable environmental conditions [9]. Such information may further reveal relationships between populations and their environment, enabling the evaluation of habitat stability and suitability, and the monitoring of ecosystem change over time [10, 11].

Gorgonian octocorals represent one of the most important benthic taxonomic groups in the Mediterranean [12]. Given the colonies’ three dimensional growth, gorgonian populations greatly modulate benthic habitats and enhance the overall structural complexity, biomass, and species diversity [13, 14]. Furthermore, they act as ecosystem engineers (*sensu* [15]) by modifying current flow, sedimentation rates and shading levels [16–18]. As several other coral species, Mediterranean gorgonians are long-lived, with slow growth, delayed maturity, low or infrequent recruitment success and reduced post-settlement survival [19–22]. The slow population dynamics of gorgonians render them susceptible to a wide range of direct or indirect anthropogenic stressors. Mechanical damage (mainly caused by fishing and unregulated recreational diving), pollution and mucilagenous algal aggregates represent localized types of disturbance [23–25], whereas biological invasions [26] and mass mortality outbreaks, related to climate induced temperature anomalies, constitute basin-scale threats [27, 28].

The yellow octocoral *Eunicella cavolini* (Koch, 1887) is one of the most common gorgonian species of the Mediterranean hard-bottom communities, and forms distinct facies within the emblematic coralligenous assemblages [14, 29]. Its distribution range is wide, although patchy in terms of abundance, and it is known to occur from the W Mediterranean and Tunisian coasts to the Aegean Sea, and the Sea of Marmara [30, 31]. Still, information regarding its distribution and ecological characteristics in different parts of the Mediterranean is lacking, while population trends and conservation status remain largely unknown. The limited number of studies on the ecology of this species are restricted in space and time [11, 29, 32–39], while more recent information comes primarily from research regarding the effects of mass mortality events (MMEs) on gorgonian species [27, 28, 40, 41]. These studies commonly underline the limitations posed by the absence of pre-disturbance data as critical baselines for the rigorous evaluation of gorgonian populations under stress [42, 43].

Given the rapid alteration of the marine environment due to direct and indirect human induced pressures [44, 45], assessment of ongoing threats and effective management decisions must be based on a thorough understanding of the natural spatial and temporal variability exhibited by species and their populations living in different geographic areas. In response to these requirements, *E*. *cavolini* populations were studied in three distinct biogeographical regions of the Mediterranean, namely the NW Mediterranean, the CE Adriatic and the N Aegean seas. Aiming to provide comparable, quantitative reference data for future monitoring and assessment of future impacts or threats, we focused on the upper depth distribution range of the species (<40 m), where populations are exposed to greater variability of environmental conditions [14, 46] and higher level of human induced disturbances [47, 48]. This study is the first to assess the population structure and disturbance levels of *E*. *cavolini* populations across most parts of its known distribution in the Mediterranean, and geographically represents the widest ranging demographic study of an octocoral in the basin.

## Materials and Methods

### Field survey

**Fig 1:**
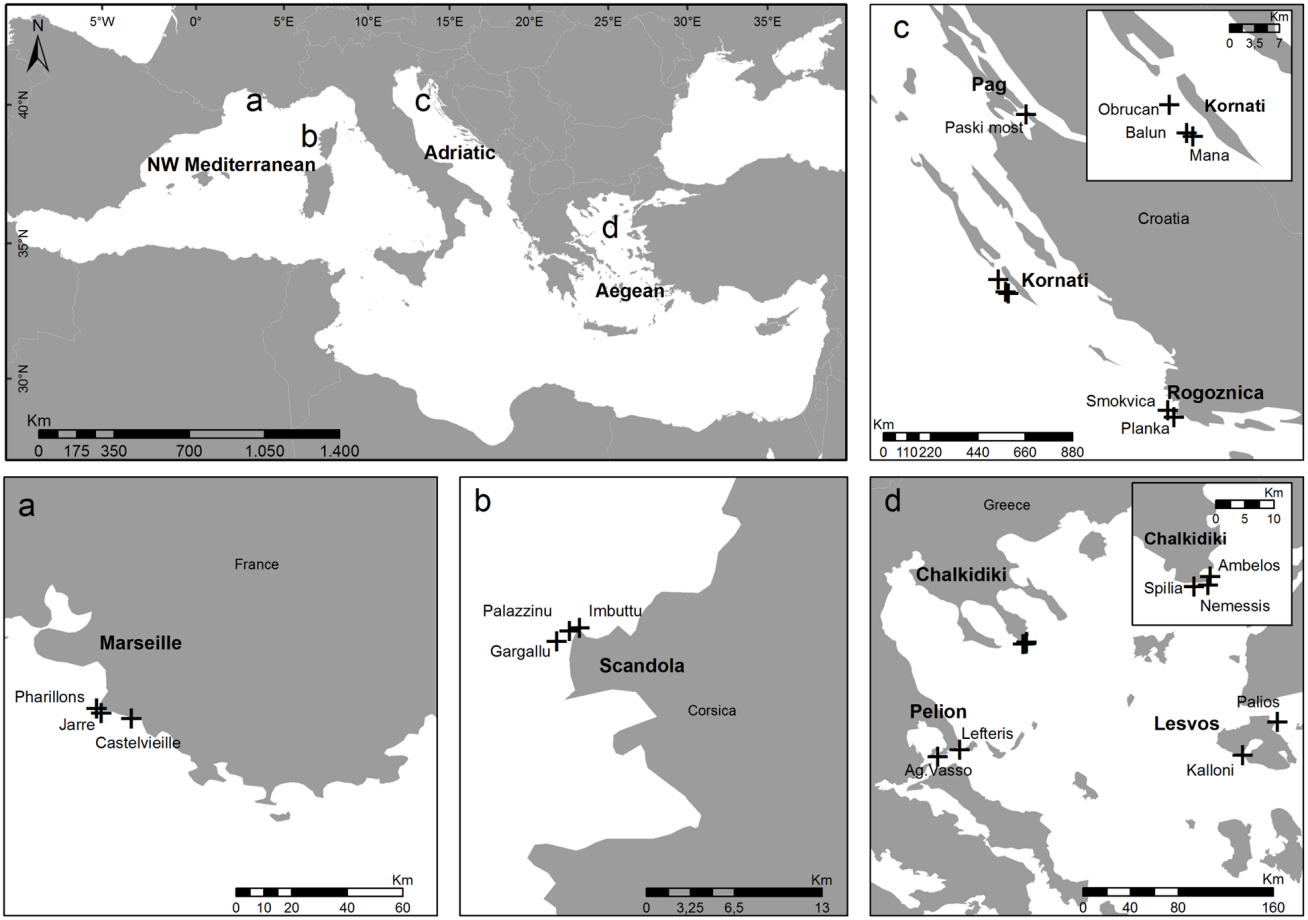
Map of the Mediterranean showing the investigated regions of the NW Mediterranean, CE Adriatic and N Aegean. Inset figures a—d present localities (in bold). Crosses mark the study sites of the yellow gorgonian *Eunicella cavolini*.

A three-level hierarchical sampling design was applied to assess the spatial patterns of *E*. *cavolini* populations, including: a) regions (located thousands of km apart), b) localities within regions (tens to hundreds of km apart), and c) sites within localities (hundreds of m to a few km apart). Three regions of the Mediterranean Sea were considered: the NW Mediterranean, CE Adriatic and N Aegean. Within each region, 2 to 3 localities were chosen, and within each locality 1 to 3 random sites were investigated. A total of 19 sites with well-developed *E*. *cavolini* populations were studied within 2 localities of the NW Mediterranean (Marseille, Scandola), 3 localities of the CE Adriatic (Kornati, Pag, Rogoznica), and 3 localities of the N Aegean (Pelio, Chalkidiki, Lesvos, Fig 1, Table 1). *In situ* underwater demographic surveys were conducted within the upper distribution depth range of the species (<40 m). Data for each population were collected once, during the period 2005–2013. Field surveys in the locality of Scandola were conducted under the authorization of the Scientific Comittee of Réserve Marine de Scandola—Parc Regional de Corse. In the case of Kornati National Park a special permission was issued by the Croatian Ministry of Culture—Department for Nature Protection. For the remaining sites, no specific permits were required at the time of field work for the sampling protocols described herein. Locations were not privately owned and the study did not involve endangered or protected species. Our study was based exclusively on non-destructive methods, and no plant or animal material was collected.

**Table 1:** Characteristics of studied sites.

Region	Locality	Site	Depth range Min—Max (m)	Coordinates		Habitat	Inclination	Protection level
NW Mediterranean	Marseille	Jarre	5–20	43°11'47"N	05°21'45"E	Wall	Vertical	MPA
		Castelvieille	5–35	43°12'01"N	05°29'39"E	Wall	Vertical	MPA
		Pharillons	10–45	43°12'27"N	05°20'18"E	Wall	Vertical	MPA
	Scandola	Imbuttu	15–45	42°22'41"N	08°33'05"E	Wall	Vertical	MPA
		Palazzinu	15–40	42°22'48"N	08°33'00"E	Wall	Vertical	MPA
		Gargallu	15–40	42°22'21"N	08°32'05"E	Wall	Sub-vertical	MPA
CE Adriatic	Kornati	Mana	5–60	43°48'01''N	15°15'59''E	Wall	Vertical	MPA; Natura 2000
		Balun	5–60	43°48'14''N	15°15'18''E	Wall	Vertical	MPA; Natura 2000
		Obrucan	5–55	43°50'11''N	15°13'12''E	Wall	Vertical	MPA; Natura 2000
	Pag	Paski most	12–35	44°19'07''N	15°15'38''E	Wall	Cascading	unprotected
	Rogoznica	Smokvica	15–50	43°30'38"N	15°56'32"E	Wall	Cascading	unprotected
		Planka	15–50	43°29'36''N	15°58'09''E	Wall	Cascading	unprotected
Aegean	Pelio	Lefteris	20–30	39°08'31"N	23°20'39"E	Rocky outcrop	Vertical	unprotected
		Ag.Vasso	30–50	39°05'08"N	23°07'48"E	Wall	Sub vertical	unprotected
	Chalkidiki	Ambelos	30–45	39°57'33"N	23°59'29"E	Wall	Sub-vertical	unprotected
		Nemessis	30–45	39°56'46"N	23°59'10"E	Wall	Sub-vertical	unprotected
		Spilia	32–50	39°56'38"N	23°57'31"E	Rocky outcrop	Sub-vertical	unprotected
	Lesvos	Kalloni	10–27	39°04'56"N	26°05'25"E	Rocky outcrop	Sub-vertical	Natura 2000
		Palios	30–44	39°19'42"N	26°26'10"E	Wall	Sub-vertical	unprotected

Depth range refers to the recorded depth distribution of the yellow gorgonian *Eunicella cavolini* population at each site. Inclination characterized as “cascading” refers to slopes intercepted by smaller vertical walls.

### Assessment of demographic characteristics and disturbance levels

For the assessment of the main population characteristics we followed the methodology proposed by Linares et al. [3]. Colonies’ density, height, proportion of injured surface and type of injury were chosen as the main population descriptors, and measurements were taken using 50×50 cm haphazardly placed quadrats within the *E*. *cavolini* populations.

Density was determined based on the number of colonies present within 50×50 cm quadrats, averaged and recalculated for 1 m^2^ surface. For each colony found, maximum height was measured as the distance from the colony base to the tip of the furthest apical branch. At each site, the aforementioned parameters were assessed over an area of more than 3 m^2^ and for more than 45 colonies, thus satisfying previously determined minimum sample size criteria [3].

Three descriptors were used to assess the impact of potential disturbances: extent of injury per colony, type of injury, and proportion of healthy colonies. The combined investigation of the type and extent of injury may provide insights regarding past disturbance events, and allow for an estimation of the approximate time of their occurrence [25, 42]. Extent of injury was estimated as the proportion of the colony’s total surface that appeared devoid of coenenchyma tissue (i.e. denuded axis) and/or that was overgrown by other organisms. Based on the presence/absence of different epibionts, and the time it takes for their development, three types of injury were identified; Type A: denuded colony axis, indicating a new injury up to 1 month; Type B: colony overgrowth by pioneer species, such as filamentous algae and hydrozoans, representing injuries of approximately 1–12 months old; Type C: colony overgrowth mostly by bryozoans, sponges and algae, reflecting an old injury of approximately ≥12 months [42]. Overall, out of the 3188 colonies measured, injury data were collected for 3045 colonies and where used to calculate mean extent of injury and percentage of healthy or affected colonies. Colonies with less than 10% of injured surface were considered as healthy, colonies with injuries ≥10% of total surface where classified as affected, whereas 100% of injury corresponded to death [3, 28]. Type of injury was quantitatively described only for colonies with ≥10% of injured surface (i.e. affected colonies); their proportion was calculated against the total number of colonies for which extent of injury was assessed per population.

### Data analysis

To assess the height frequency distribution per site, height measurements of colonies displaying <100% of injured surface were grouped into five classes: 1–10, 11–20, 21–30, 31–40, and >41 cm, and the descriptive distribution parameters of skewness (g1) and kurtosis (g2) were estimated. Coefficients of g1 and g2 were considered significant if the ratio to their standard error was >2 [49]. The relation between mean height and density was explored using a Spearman rank order correlation.

A non-metric multidimensional scaling (MDS) ordination [50] was performed to visualize patterns of population structure based on the following parameters after normalization of data: mean height, max height, proportion of the smaller (0–20 cm) and larger (>30 cm) height classes, density and upper depth distribution limit. One-way, non-parametric analysis of variance PERMANOVA [51], based on square root transformed data and Euclidean distances, was used to test for spatial variability in colony height and density. A three-factor hierarchical design was applied using "region" (3 levels) as a fixed factor, "locality" (8 levels) as a random factor nested within region, and "site" (19 levels) as a random factor nested within locality. Pair-wise comparisons were performed to determine specific inter or intra-regional differences when the main test indicated significant differences. Significance was confirmed based on 9999 permutations. Analyses were performed using the PRIMER v6 software with PERMANOVA+ add-on package [52, 53].

## Results

### Upper depth distribution range

**Fig 2:**
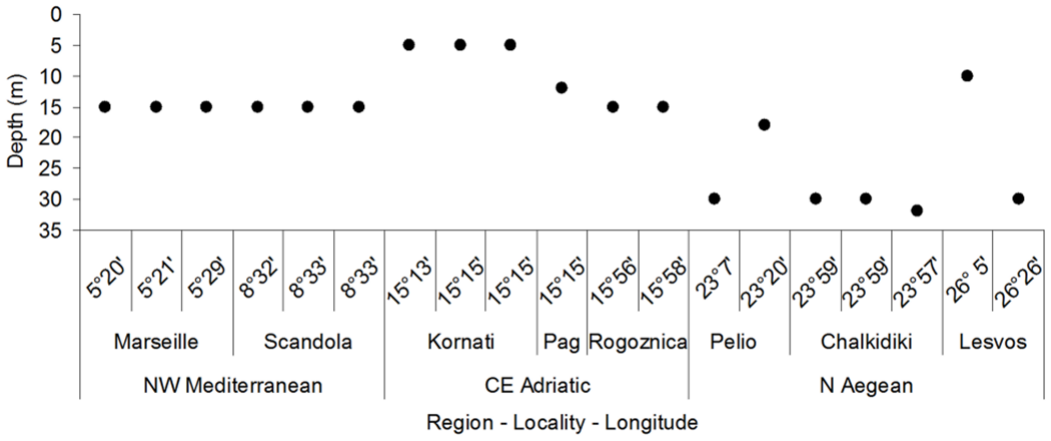
Upper depth distribution limits for the investigated *Eunicella cavolini* populations along a longitudinal gradient.

The upper depth distribution limit varied considerably along the longitudinal gradient (Fig 2). In all localities of the NW Mediterranean and CE Adriatic, the upper distribution limit of *E*. *cavolini* populations was found at 15 m depth or shallower, with some populations appearing at 5 m depth in the locality of Kornati (CE Adriatic). On the contrary, most populations of the N Aegean were found in waters deeper than 30 meters, with the exception of two populations that were located at 10 and 20 m depth.

### Demographic characteristics

**Fig 3:**
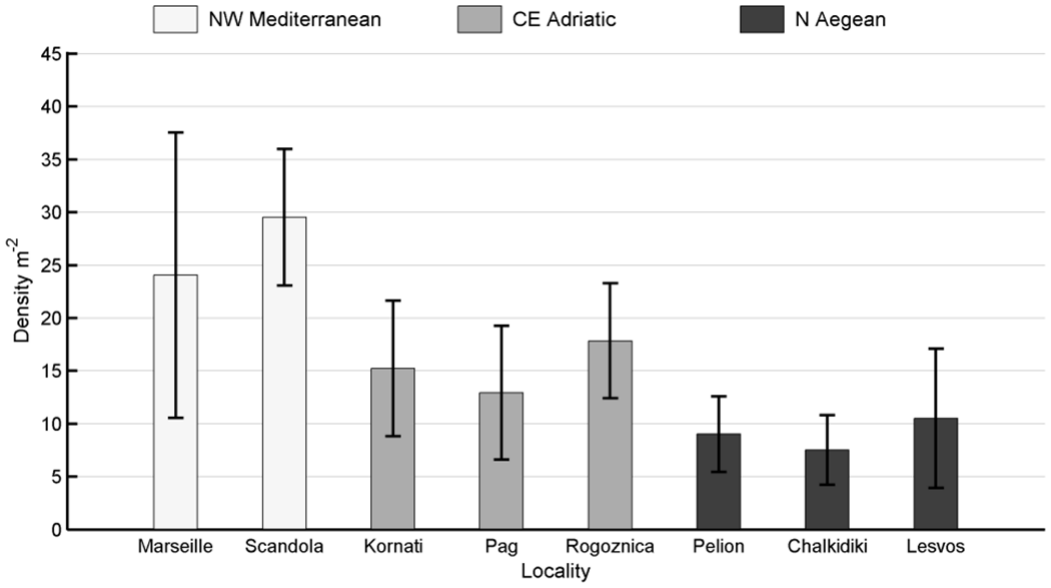
Mean density (colonies per m^2^) of *Eunicella cavolini* populations, tabulated by locality. Whisker span indicates standard deviation. Colors denote geographic regions.

Mean population density of *E*. *cavolini* was 14.9 ± 14.6 SD (standard deviation) per m^2^. The minimum density of 4.5 colonies per m^2^ was observed at Ambellos (N Aegean), while the maximum density of 62 colonies per m^2^ was recorded at Jarre (NW Mediterranean, Table 2). Among localities (Fig 3), higher densities were recorded in Scandola (29.5 ± 12.9 SD per m^2^) and Marseille (24.1 ± 27 SD per m^2^) within the NW Mediterranean, followed by Rogoznica (17.8 ± 10.9 SD per m^2^), Kornati (15.2 ± 12.8 SD per m^2^), and Pag (12.9 ± 12.6 SD per m^2^) within the CE Adriatic, while the lowest densities were observed in the localities of the N Aegean, namely Lesvos (10.5 ± 13.1 SD per m^2^), Pelio (9.03 ± 7.1 SD per m^2^), and Chalkidiki (7.5 ± 6.6 SD per m^2^). PERMANOVA results suggest that a significant variability in density exists among regions and sites (p < 0.05), but not between localities (Table 3). Furthermore, the greatest variation in density, indicated by the components of variation, was observed among regions. The pair-wise tests show that a significant difference in population density exists among all regions (p < 0.05, Table 4) and between sites in 4 of the 7 localities analysed (Table A in S1 File).

**Table 2:** Population characteristics of *Eunicella cavolini* per region, locality and site.

Region	Locality	Site	Sampling depth (m)	Area (m^-2^)	Density (m^-2^)	Height (cm)							
					Mean	N	Mean	Min	Max	SD	SE	Skewness	Kurtosis
NW Mediterranean	Marseille	Jarre	15–20	3.50	62.00	217	12.21	1.0	38.0	7.22	0.49	0.69*	0.20
		Castelvieille	15–20	6.00	12.83	76	18.28	3.0	45.0	9.21	1.06	0.49	-0.05
		Pharillons	15–20	4.75	10.32	49	14.20	2.0	35.0	7.79	1.11	1.02*	0.73
	Scandola	Imbuttu	15–20	9.50	29.47	280	13.76	1.5	28.5	5.32	0.32	0.07	-0.47
		Palazzinu	20–23	9.00	30.78	270	12.33	0.5	37.0	5.96	0.36	0.68*	0.73*
		Gargallu	20–25	8.75	28.34	245	15.12	2.0	33.0	5.72	0.36	0.29	-0.01
CE Adriatic	Kornati	Mana	9–28	16.75	12.24	198	10.15	1.5	43.0	4.64	0.33	1.89*	11.67*
		Balun	13–28	20.25	17.43	345	10.92	1.0	28.0	5.03	0.27	0.39*	0.08
		Obrucan	15–28	23.50	15.45	360	15.29	2.0	45.0	8.27	0.43	0.45*	-0.27
	Pag	Paski most	20–30	7.50	12.93	89	19.47	3.0	50.0	10.04	1.06	0.85*	0.18
	Rogoznica	Smokvica	20–30	7.50	17.06	126	20.66	5.0	50.0	8.43	0.75	0.72*	0.79
		Planka	23–30	5.50	18.91	102	17.40	4.0	50.0	10.12	1.00	1.08*	0.66
N Aegean	Pelio	Lefteris	24–30	11.00	10.36	113	17.13	4.0	53.0	10.29	0.97	1.13*	1.09*
		Ag.Vasso	32–35	12.25	7.84	95	23.66	2.0	62.0	12.08	1.24	0.37	-0.25
	Chalkidiki	Ambelos	30–40	26.25	4.46	114	25.06	5.0	50.0	9.16	0.86	0.21	0.03
		Nemessis	30–38	9.25	11.35	105	13.81	2.0	34.0	6.84	0.67	0.44	-0.44
		Spilia	35–40	7.75	13.29	103	23.17	3.0	51.0	10.93	1.08	0.21	-0.56
	Lesvos	Kalloni	13–27	6.25	23.52	127	16.71	1.0	46.0	11.11	0.98	0.49*	-0.45
		Palios	33–40	18.00	6.00	108	23.86	3.0	48.0	10.62	1.02	0.07	-0.88

*statistically significant results.

**Table 3:** Summary of PERMANOVA results for *Eunicella cavolini* population density.

	Source of variation					
	df	SS	MS	Pseudo-F	P(perm.)	Unique perm.
Region	2	726.65	363.33	21.237	0.0008*	9968
Locality(Region)	5	57.722	11.544	0.31125	0.8763	9960
Site(Locality(Region))	11	409.2	37.2	14.303	0.0001*	9944
Residuals	834	2169.1	2.6008			
Total	852	3362.7				

Tests of significance were run based on Euclidean distances for square root transformed data.

*statistically significant differences (*p*<0.05).

**Table 4:** Summary of PERMANOVA pairwise comparisons for *Eunicella cavolini* population density among regions.

Pairwise test for regions’ density	t	P(perm.)	Unique perm.
NW Mediterranean, CE Adriatic	2.9077	0.0183*	9970
NW Mediterranean, Aegean	7.0213	0.0019*	9965
CE Adriatic, Aegean	4.263	0.0114*	9965

Tests of significance were run based on Euclidean distances for square root transformed data.

*statistically significant differences (*p*<0.05).

**Fig 4:**
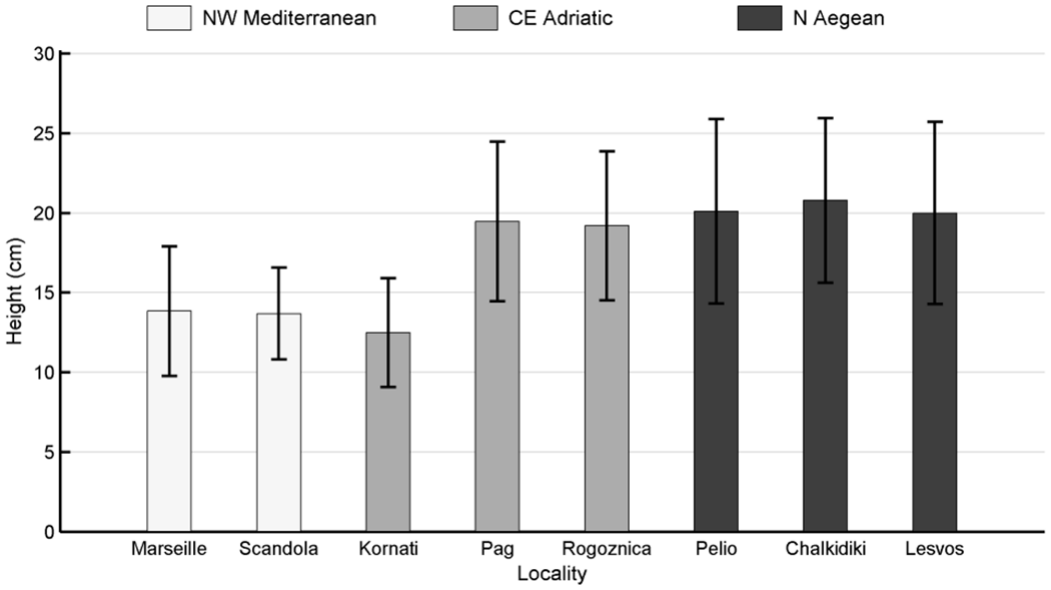
Mean height of *Eunicella cavolini* colonies per locality. Whisker span indicates standard deviation. Colors denote geographic regions.

Overall, mean colony height was 15.6 ± 8.9 SD cm, while the maximum recorded height was 62 cm (Table 2). Among localities (Fig 4), mean colony height was greater at Chalkidiki (20.8 ± 10.3 SD cm), Pelio (20.1 ± 11.6 SD cm) and Lesvos (19.9 ± 11.4 SD cm) in the N Aegean, while equally high values were recorded at Pag (19.5 ± 10 SD cm) and Rogoznica (19.2 ± 9.3 SD cm) in the CE Adriatic. Lower height values were observed at Marseille (13.8 ± 8.1 SD cm) and Scandola (13.7 ± 5.8 SD cm) in the NW Mediterranean, as well as Kornati (12.5 ± 6.8 SD cm) in the CE Adriatic. Significant differences in height were only found at the level of sites within localities (PERMANOVA test, Table 5; Table B in S1 File).

**Table 5:** Summary of PERMANOVA results for *Eunicella cavolini* colony height.

Colony height	Source of variation					
Source	df	SS	MS	Pseudo-F	P(perm.)	Unique perm.
Region	2	295.97	147.98	2.4786	0.1657	9955
Locality(Region)	5	176.86	35.371	1.4701	0.2791	9955
Site(Locality(Region))	11	326.65	29.695	29.73	0.0001*	9932
Residuals	3103	3099.4	0.99884			
Total	3121	3898.9				

Tests of significance were run based on Euclidean distances for square root transformed data.

*statistically significant differences (*p*<0.05).

**Fig 5:**
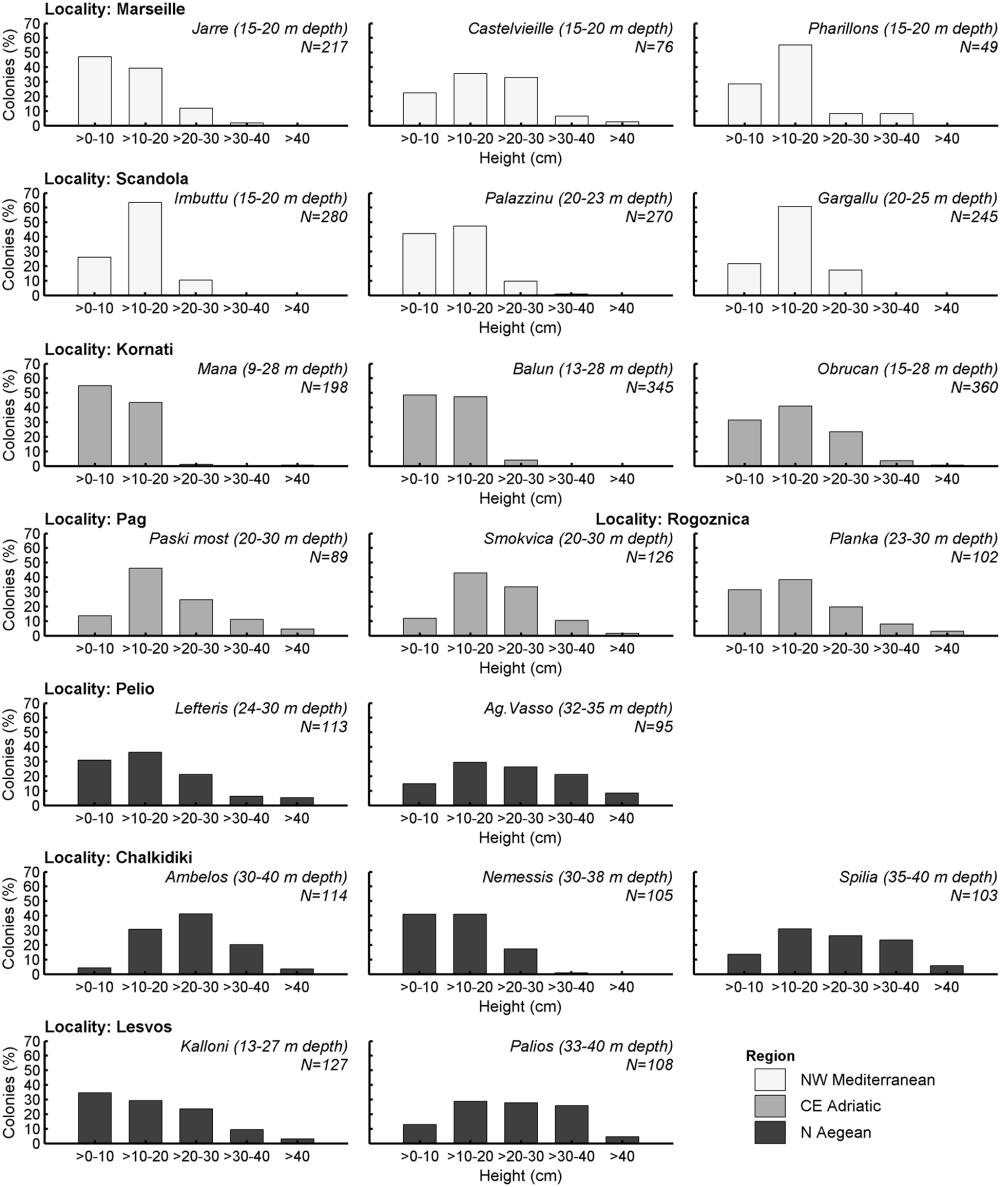
Height frequency distribution of *Eunicella cavolini* populations per site, grouped by locality (in bold) and region (different colors). Italics indicate site name, numbers in parentheses denote sampling depth range, and (N) corresponds to the number of colonies used.

Height frequency distribution of *E*. *cavolini* populations appeared to be either positively skewed or relatively symmetric depending on site (Fig 5). A significant positive skewness, indicating a prevalence of the smaller height classes, was found for the populations of Jarre, Pharillons, and Palazzinu in NW Mediterranean, all populations of the CE Adriatic, and for the shallower sites in the N Aegean, namely Lefteris and Kalloni (Table 2). Of these positively skewed populations, Palazzinu, Mana, and Lefteris displayed additionally a significant positive kurtosis value, suggesting a dominance of either one or both of the two smaller height classes (>0–10 and >10–20 cm). The majority of the N Aegean populations presented a non-significant negative kurtosis value. Although an important proportion of large colonies (20–30 cm) appeared in most sites, the number of colonies with height >30 cm was generally low, and more pronounced in certain sites of the CE Adriatic and N Aegean.

**Fig 6:**
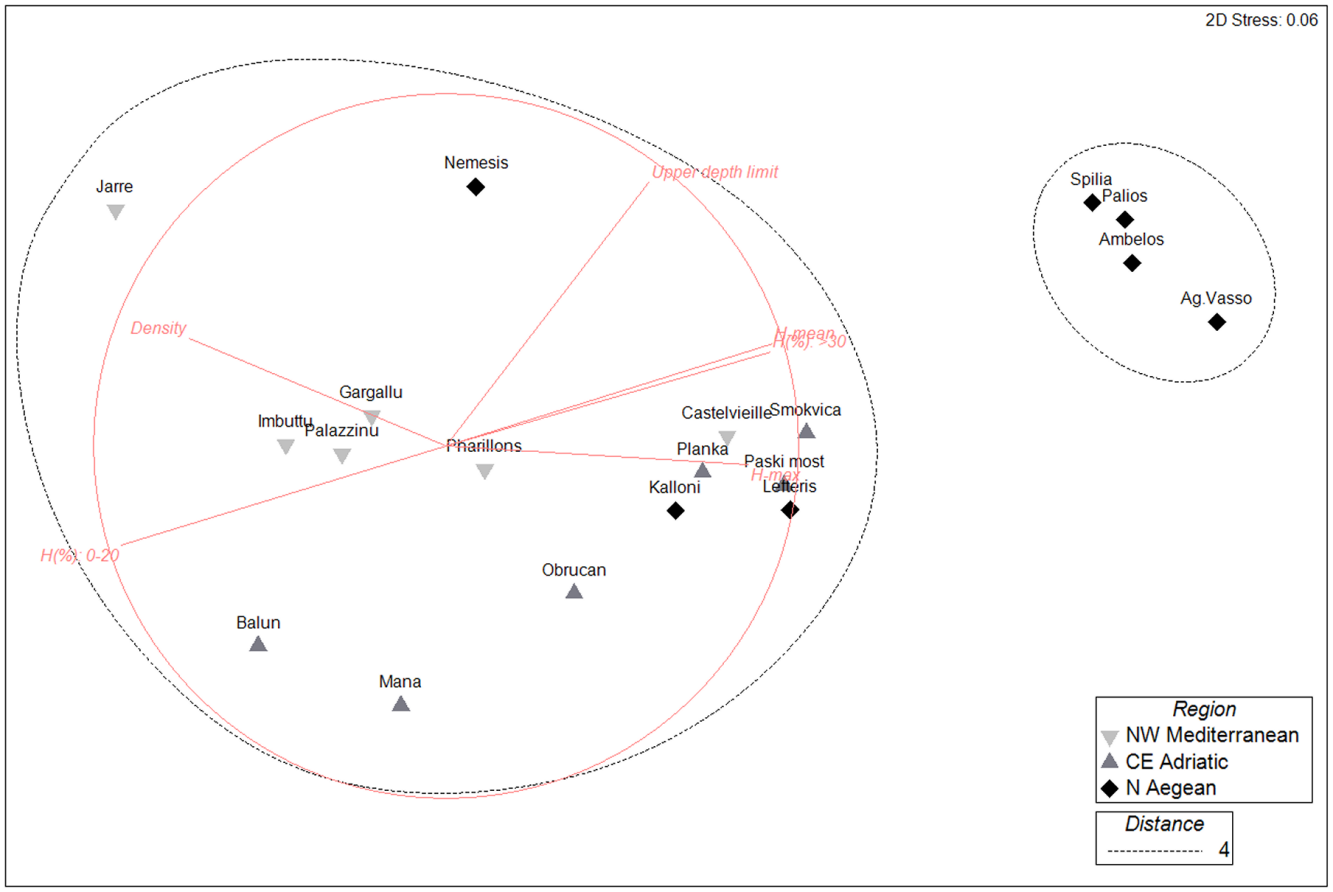
Non-metric multidimensional scaling (MDS) plot of *Eunicella cavolini* population structure per site. Different markers indicate different regions. Black dotted lines signify clusters formed at Euclidean distance equal to 4. The superimposed red lines denote the Euclidean distance coefficients used, after data normalization: mean height, max height, H% (proportion of height classes ≤20 cm and >30 cm), height skewness, height kurtosis, density, and upper depth distribution.

According to the Spearman rank order correlation values of density and mean height across sites displayed a significant inverse relation (r_s_ = -0.58, p = 0.009). The MDS plot of *E*. *cavolini* populations according to basic demographic characteristics (Fig 6) produced two main clusters at a stress level of 0.06, indicating good ordination; one cluster encompassing all NW Mediteranean and CE Adriatic populations, as well as the shallower populations of the N Aegean, and a second one including the deeper populations of the N Aegean.

### Disturbance levels

Among populations, mean extent of injury of gorgonian tissue varied substantially, ranging from 0.8% to 38.3% (Table 6). Most populations (14 out of 19) presented a mean extent of injury less than 10%, while higher values were observed at Planka (13%), Smokvica (16.5%) and Paski most (25.8%) in the CE Adriatic, and at Ambelos (18.6%) and Kalloni (38.3%) in the N Aegean. The proportion of injured colonies (i.e. ≥10% to ≤99% of injured surface) ranged between 0–64%, with highest values at Paski most (37.1%) and Smokvica (39.8%) in the CE Adriatic, and Ambellos (64.1%) and Kalloni (52.4%) in the N Aegean (Table 6). The percentage of dead colonies was generally below 3.5% for the majority of sites, with the exception of Paski most (8.25%) and Kalloni (13.6%). Overall, the greatest proportions of healthy colonies (<10% of injured surface) were recorded in the localities of Marseille (75.3–100%), Scandola (83–91%) and Kornati (78.5–82.6%) (Table 6).

**Table 6:** Summary data on injury characteristics of *Eunicella cavolini* colonies per site.

					Extent of colony injury (%)		Proportion of uninjured, injured and dead colonies			Proportion of coloniesper type of injury		
Region	Locality	Site	Year	N	Mean	SD	<10%	≥10–≤99%	100%	A	B	C
NW Mediterranean	Marseille	Jarre	2005	75	6.7	10.5	85.33	14.67	0.00	10.66	2.67	9.33
		Castelvieille	2005	77	9.6	22.4	75.32	23.38	1.30	0.00	1.30	23.38
		Pharillons	2005	49	0.8	1.9	100.00	0.00	0.00	0.00	0.00	0.00
	Scandola	Imbuttu	2013	280	3.1	10.3	89.29	10.71	0.00	0.00	0.00	10.71
		Palazzinu	2013	277	7.9	21.7	83.03	14.44	2.53	0.00	0.00	16.97
		Gargallu	2013	248	3.3	14.2	91.94	6.85	1.21	0.00	0.00	8.06
CE Adriatic	Kornati	Mana	2009	205	7.9	20.7	78.54	18.05	3.41	1.46	8.78	11.71
		Balun	2009	353	5.5	18.4	89.52	8.22	2.27	0.28	1.13	9.35
		Obrucan	2009	362	5.7	15.8	82.60	16.57	0.83	0.28	3.31	13.81
	Pag	Paski most	2011	97	25.8	36	54.64	37.11	8.25	1.03	10.31	37.11
	Rogoznica	Smokvica	2009	128	16.5	26.3	58.59	39.84	1.56	0.00	1.56	38.28
		Planka	2009	104	13.3	24.2	66.35	31.73	1.92	0.00	0.00	33.65
N Aegean	Pelio	Lefteris	2011	114	8.5	16	64.91	34.21	0.85	0.00	26.32	6.14
		Ag.Vasso	2011	96	6.7	16.3	77.1	21.87	1.04	0.00	17.71	3.13
	Chalkidiki	Ambelos	2011	117	18.6	24.9	33.33	64.1	2.56	46.15	11.97	5.98
		Nemessis	2011	105	4.1	12.1	85.71	14.29	0.00	0.00	14.29	0.00
		Spilia	2011	103	6.9	15.7	78.6	21.4	0.00	0.00	19.42	1.91
	Lesvos	Kalloni	2013	147	38.3	39.1	34.01	52.38	13.61	12.24	57.82	49.66
		Palios	2011	108	2.5	8.3	89.81	10.18	0.00	0.93	9.26	0.93

Proportion of colonies per type of injury was estimated using only colonies displaying ≥10% of injured surface against the total number of colonies per site.

The majority of affected colonies (≥10% of injured surface) in the NW Mediterranean and the CE Adriatic presented type C (old) injuries. In the N Aegean, populations mainly presented overgrowth by pioneer species (type B injury); exceptions were Ambelos, which displayed high proportion of recent tissue necrosis (type A injury: 46.15%), and Kalloni which had high levels of all types of injury. As the time of sampling varied among localities, no statistical comparisons regarding disturbance parameters were attempted.

## Discussion

The extensive geographic distribution of *E*. *cavolini* and its relatively wide bathymetric range (<10–220 m [30, 35, 54, 55]) reflect its ability to adapt and survive over highly variable abiotic conditions. In this study, we carried out a comprehensive analysis of *E*. *cavolini* population structure and dynamics at three distinct Mediterranean regions, in order to facilitate a better understanding of the patterns observed across contrasting environmental gradients.

### Upper depth distribution range

One of the most robust findings is the deeper upper bathymetric limit of populations in the N Aegean, compared to those thriving in the NW Mediterranean and CE Adriatic. In the latter regions, the upper bathymetric limit of *E*. *cavolini* was at 5–15 m. These observations are in line with the minimum depths reported in other localities of the western Mediterranean basin (e.g. [29, 35, 38, 56]) or the Adriatic Sea (e.g. [57]), and the fact that the species can withstand a fairly wide range of light intensity (1–44% of surface light according to [29]). Within the N Aegean, most populations were located at depths below 30 m. According to previous reports, gorgonian species in the Aegean Sea are generally observed in waters deeper than 40 m [58, 59] and are rarely found at depths shallower than 20 m [60, 61].

We hypothesize that the observed distribution patterns are related to the variability of abiotic factors that predominate in the distinct regions under study, putatively coupled with biotic interactions [62, 63]. In fact, Zabala & Ballesteros [62] suggested that in oligotrophic areas suspension feeders are restricted to deeper waters or in areas were strong currents prevail, while in shallow waters algal species can outcompete long-lived suspension feeders, such as gorgonian species. Moreover, Mediterranean gorgonian species are particularly vulnerable to temperature anomalies, which are more likely to occur in shallow waters. According to the above, the downward shift of the upper bathymetric limit observed in the N Aegean may be due to the more oligotrophic conditions and higher water temperatures characterizing this region compared to those of the NW Mediterranean and CE Adriatic [64, 65]. The only exceptions to the bathymetric pattern of the N Aegean were the sites of Kalloni (10 m depth) and Lefteris (20 m depth), which are characterized by the presence of strong currents that are known to promote the growth of suspension feeders such as *E*. *cavolini* [16].

### Population structure and dynamics

*E*. *cavolini* population density ranged from 4.46 to 62 colonies per m^2^, although the species may attain much greater densities [39]. Density was highly variable across regions, and displayed a decreasing trend from west to east. Highest values were observed in the NW Mediterranean, intermediate in the CE Adriatic, and lowest in the N Aegean, while in all regions significant differences were also detected at the level of sites within localities. Variability in density among sites within the same depth range (i.e. <40 m) has also been observed in other localities of the NW Mediterranean (e.g. for *E*. *cavolini* [35, 39, 40]), as well as for other Mediterranean gorgonians (e.g. for *E*. *singularis* [3, 66], *Paramuricea clavata* [3, 67], and *Corallium rubrum* [7, 68]). According to these studies, density is usually related to factors that affect reproduction and recruitment success. For example, Weinbauer & Velimirov [11, 39] justified the wide density differences observed in *E*. *cavolini* populations (15–180 colonies per m^2^) among nearby sites at the Bay of Calvi—Corsica, on the basis of substrate availability, turbulence, abundance of large colonies and degree of colony overgrowth by other organisms. They further related these factors to the successful reproduction, settlement and survival of new colonies. On the other hand, increased densities have also been observed during periods of population recovery from MMEs, once detachment of dead colonies created free space for new recruits (e.g. [69–71]). In the present study, density was overall inversly related to height, supporting the idea that recruitment is driven by intra-specific competition mechanisms, where the lack of large colonies enhances recruitment success.

**Fig 7:**
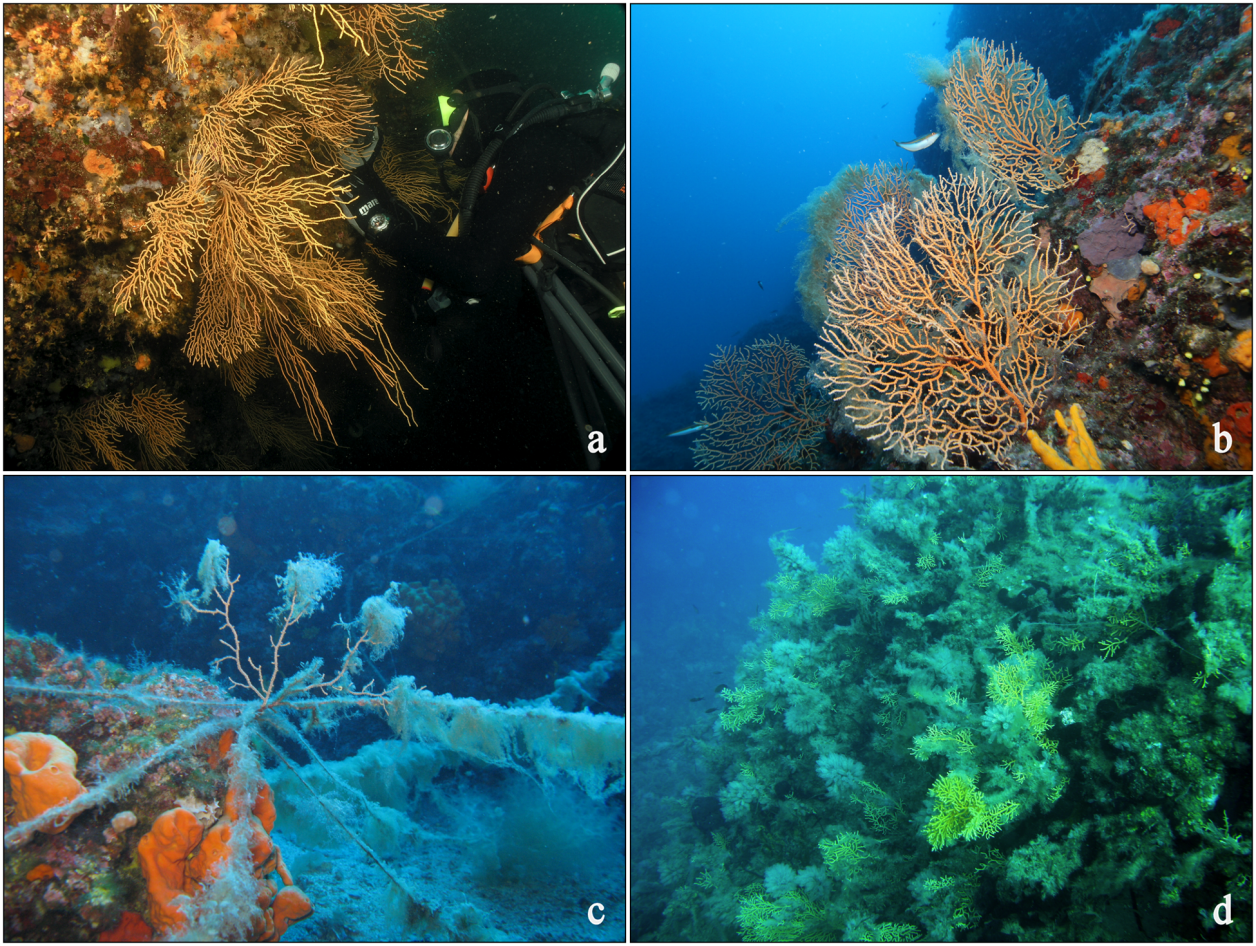
Colonies of the yellow gorgonian *Eunicella cavolini* exposed to different orientations and disturbances. a) in a large overhang; b) on open rock; c) under stress by fishing gear and mucilagenous algae, d) heavily overgrown during a mass mortality event. Photos a and b are courtesy of Thanos Dalianis and Panagiotis Papadelis, respectively.

With regard to colony height, mean value for all examined populations was 15.7 ± 8.87 cm, with a maximum of 62 cm. Similar height values have been reported for both shallow [35] and deep water populations of *E*. *cavolini* (i.e. >70 m, [54]). As in several other octocoral species, *E*. *cavolini* is known to diplay a high level of phenotypic plasticity (Fig 7A and 7B) by modulating its structural characteristics and growth form (i.e. including fan size, shape, orientation, and polyp number) in response to water movement [32, 34, 37, 72]. It is therefore possible that the direction and velocity of prevailing water currents are the main factors determining colony height in several sites of the present study. In this respect, the greater proportion of larger colonies (>30 cm) found in the deeper sites of the CE Adriatic and N Aegean may be due to the greater environmental stability of deeper waters [4, 46], and the decrease of hydrodynamic forces that are known to affect colony morphology. At the same time, the greater proportion of small colonies (<20 cm) observed in sites characterised by higher densities (mainly in the NW Mediterranean) may be indicative of a more dynamic environment (in terms of water flow and productivity), which reduces the optimal size of colonies, but retains highly reproductive, small sized colonies belonging to a wide range of age classes [73].

Although morphological plasticity may partly explain differences in population structure among sites, no significant differences were detected in colony height at the level of localities or regions. The overall patterns of height frequency distribution suggest that *E*. *cavolini* populations were at varying stages of development, driven by different recruitment and mortality dynamics. NW Mediterranean and CE Adriatic populations were mainly characterised by the predominance of one or both of the smaller size classes (0–10, >10–20 cm), indicating either continuous or pulse recruitment episodes. In the majority of the N Aegean populations, all size classes were almost equally represented, while the number of small colonies (especially of the 0–10 cm height class) was typically low, suggesting limited recruitment. Still, the presumably low recruitment dynamics of the N Aegean populations may be compensated by the presence of a large number of mature colonies during sporadic reproductive events, since gamete production, and hence reproductive output, increase exponentially with colony size [22, 74–76]. On the contrary, the low abundance of large colonies, or even the lack of them, observed in several studied sites across regions, may be indicative either of newly formed and expanding populations, or of the existence of additional natural and/or human-induced pressures (e.g. MMEs, mechanical damage especially caused by unregulated fisheries and recreational marine activities, or extreme wave action) which particularly affect the survivorship of larger height classes [7, 10, 39, 42, 43, 69, 77].

Patterns of height frequency distribution similar to those observed herein have been documented for several gorgonian populations in other localities of NW Mediterranean and E Adriatic. A unimodal peak at the second size class—implying periodic recruitment—has been observed in populations of *E*. *cavolini* dwelling both shallow (<30 m, NW Corsica [11]) and deep waters (>70 m, S Tyrrhenian Sea [54]), in shallow *P*. *clavata* populations off the Spanish and French coasts (<40 m [2, 3]), as well as in deep *E*. *singularis* populations (>50 m, off the Spanish coasts [4]). Positively skewed populations, displaying prevalence of new recruits (0–10 cm) and low proportion of larger gorgonians, have been described for shallow *E*. *singularis* populations along the Spanish coasts (<25 m [3, 4]). Yet again, several authors [77, 78] have reported similar population structure for *P*. *clavata* in the Ligurian Sea following MMEs. On the other hand, populations characterised by a predominance of the smallest height class along with a high proportion of large colonies—reflecting well structured populations with continuous recruitment—were not observed during the present study, regardless the wide distribution range covered. Occasional reports of the latter pattern at depths <50 m include a single population of *E*. *cavolini* in NW Corsica [11] and *P*. *clavata* populations in the E Adriatic [79].

### Extent of injuries: baseline for future monitoring

Injuries of gorgonian colonies are either caused by mechanical abrasion and predation or through necrosis of living tissue under stress conditions (e.g. persistence of thermal anomalies) causing partial and/or total mortality of colonies (Fig 7C and 7D). Injured parts may break, regenerate or become colonised by overgrowing organisms [36, 67]. *E*. *cavolini* is particularly vulnerable to such injuries, and has been affected by several MMEs related to temperature anomalies in the NW Mediterranean, some of which have led to substantial population reductions (e.g. [27, 28, 41]). Although injury estimates were taken over different years in this study, thus not allowing for direct quantitative comparisons among regions, they do however provide insights regarding conservation status at the time of assessment, as well as baseline data for future assessments. Our assessment in Marseille and Scandola was realized two and ten years respectively, after one of the largest and best documented MME in the NW Mediterranean, which took place during late summer of 2003 [28]. At the time of the MME, the estimated proportion of affected *E*. *cavolini* colonies ranged between 3–50.8% (mean: 14.5 ± 14.5) in Marseille and 4.9–34.2% (mean 17.1 ± 10.6) in Scandola [28]. It is therefore possible that the number of affected colonies displaying old overgrowth (type C injury) in Marseille reflects the effects of the past MME given the slow recovery capacity of gorgonian species [22, 42]. This hypothesis is further supported by the high densities observed in the majority of the NW Mediterranean sites, and the low number of large colonies (>30 cm), as observed in other populations during recovery (e.g. [69–71]).

Within the CE Adriatic, *E*. *cavolini* populations found in the relative pristine conditions of Kornati presented a smaller mean extent of injury, as well as smaller proportion of affected colonies, compared to the populations of Pag and Rogoznica which are located more closely to the mainland and are potentially more exposed to human-induced stressors. As overgrowth by epibionts in all sites of the CE Adriatic was mostly old (injury type C), no recent disturbances were indicated at the time of assessment. Our observations are in agreement with reported disturbance values for populations of the red gorgonian *P*. *clavata* in the region [79].

Regardless locality, the majority of N Aegean populations displayed a relatively low mean extent of injury, but overall high proportion of affected colonies. The injury values observed, combined with the generally low density and recruitment success recorded, render gorgonian populations of the N Aegean more prone to potential threats. Furthermore, in the sites of Ambelos and Kalloni, proportion of injured colonies (64.1% and 52.4% respectively) suggest increased levels of stress, while the high number of dead colonies (13.6%) in Kalloni is indicative of a strong impact. Although the reasons of the observed disturbance cannot be readily addressed through the present study, the increased levels of fishing activities (especially recreational and artisanal), and recurrent periods of high nutrient loads in the wider area [80–81], constitute some of the potential contributing stressors, especially given the shallow depth range of this population.

### Closing remarks

Quantifying the demographic characteristics and disturbance levels of *E*. *cavolini* populations enabled the assessment of their conservation status and the acquisition of comparative information over a wide range of the species’ known spatial distribution [30]. The patterns observed provide insights as to how biotic, abiotic, and anthropogenic factors may affect the structure and dynamics of populations at the spatial scales addressed in this study, and subsequently influence their adaptive capacity to environmental change. Given the widespread distribution of *E*. *cavolini*, further research will allow a more comprehensive view of the population trends presented herein, and enhance understanding and mitigation of potential future impacts. Extending the same kind of approaches to include other areas and species would provide key information that will help develop effective management plans for the conservation of valuable hard-bottom communities, including coralligenous assemblages, across the highly heterogeneous Mediterranean basin [82].

## Supporting Information

S1 File Pairwise comparisons of *Eunicella cavolini* population density and colony height at different sites. (PDF)

S2 File Analytical data tables on *Eunicella cavolini* population characteristics across sites, localities and regions. (XLS)
