# Supplementary material for: The Yellow Gorgonian Eunicella cavolini: Demography and Disturbance Levels across the Mediterranean Sea
Source: PLoS One. 2015 May 5;10(5):e0126253. doi: 10.1371/journal.pone.0126253 (PMC4420262; doi:10.1371/journal.pone.0126253)
Supplement: S1 File — (PDF) [file pone.0126253.s001.pdf]

S1 File. Pairwise comparisons of *Eunicella cavolini* population density and colony height at different sites.

Table A: Pairwise comparisons of *Eunicella cavolini* population density between different sites.

Density					
**Region**	**Locality**	**Sites**	**t**	**p-value**	**Unique perms**
NW Mediterranean	Marseille	Jarre, Castelvieille	8.2771	0.0001*	9234
		Jarre, Pharillons	8.491	0.0001*	8969
		Castelvieille, Pharillons	1.0518	0.3025	2836
NW Mediterranean	Scandola	Palazzinu, Gargallu	0.82342	0.4139	9815
		Palazzinu, Imbuttu	0.4661	0.6432	9811
		Gargallu, Imbuttu	0.36442	0.7144	9831
CE Adriatic	Kornati	Obrucan, Balun	1.1748	0.241	9834
		Obrucan, Mana	1.4715	0.1461	9793
		Balun, Mana	2.5824	0.0124*	9806
CE Adriatic	Rogoznica	Smokvica, Planka	0.12852	0.8986	8611
N Aegean	Pelio	Ag.Vasso, Lefteris	0.99359	0.3308	6946
N Aegean	Chalkidiki	Ambelos, Nemesis	4.7324	0.0001*	5087
		Ambelos, Spilia	7.6613	0.0001*	3348
		Nemesis, Spilia	1.8253	0.0688	6718
N Aegean	Lesvos	Palios, Kalloni	4.9877	0.0001*	6517

*statistically significant difference (p<0.05).

Table B: Pairwise comparisons of *Eunicella cavolini* colony height between different sites.

Height					
**Region**	**Locality**	**Sites**	**t**	**p-value**	**Unique perms**
NW Mediterranean	Marseille	Jarre, Castelvieille	5.5745	0.0001*	9815
		Jarre, Pharillons	1.8146	0. 0712	9822
		Castelvieille, Pharillons	2.5223	0.0126*	9846
NW Mediterranean	Scandola	Imbuttu, Palazzinu	3.2787	0.0012*	9825
		Imbuttu, Gargallu	2.7148	0.0074*	9836
		Palazzinu, Gargallu	5.6487	0.0001*	9844
CE Adriatic	Kornati	Mana, Balun	1.4603	0.1468	9824
		Mana, Obrucan	7.3821	0.0001*	9824
		Balun, Obrucan	7.4461	0.0001*	9835
CE Adriatic	Rogoznica	Smokvica, Planka	3.1893	0.0018*	9848
N Aegean	Pelio	Ag.Vasso, Lefteris	4.1543	0.0001*	9831
N Aegean	Chalkidiki	Ambelos, Nemesis	10.24	0.0001*	9844
		Ambelos, Spilia	1.7248	0. 0914	9822
		Nemesis, Spilia	7.0881	0.0001*	9842
N Aegean	Lesvos	Palios, Kalloni	5. 299	0.0001*	9821

*statistically significant differences (p<0.05).
